# *R*-Ferrite-type barium cobalt stannate, BaCo_2_Sn_4_O_11_

**DOI:** 10.1107/S1600536808030572

**Published:** 2008-09-27

**Authors:** Hisanori Yamane, Takeshi Ono, Takahiro Yamada

**Affiliations:** a Institute of Multidisciplinary Research for Advanced Materials, Tohoku University, 2-1-1 Katahira, Aobaku, Sendai 980-8577, Japan; b Center for Interdisciplinary Research, Tohoku University, Aramaki Aza-Aoba, Aobaku, Sendai 980-8578, Japan

## Abstract

BaCo_2_Sn_4_O_11_ is isotypic with *R*-ferrite, BaTi_2_Fe_4_O_11_. The Co atoms fully occupy trigonal–bipyramidal sites (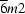) and are disordered with Sn atoms in octa­hedral sites (.2/*m* symmetry), as represented in the formula BaCoSn_2_(Co_0.34_Sn_0.66_)_4_O_11_. Ba atoms are situated in a 12-fold coordinated site (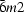 symmetry).

## Related literature

For reports on *R*-ferrite structures, BaTi_2_Fe_4_O_11_, see: Haberey & Velicescu (1974 ▶); Obradors *et al.* (1983 ▶); Cadée & Ijdo (1984 ▶); Sosnowska *et al.* (1996 ▶). For reports on R-ferrite structure with other compositions, see: Cadée & Ijdo (1984 ▶); Kanke *et al.* (1992 ▶); Martínez *et al.* (1993 ▶); Foo *et al.* (2006 ▶). Sosnowska *et al.* (1996 ▶). For Ba_3_SnCo_10_O_20_, another phase in the Ba–Co–Sn–O system, see: Sonne & Müller-Buschbaum (1993 ▶).

## Experimental

### 

#### Crystal data

BaCo_2_Sn_4_O_11_
*M* *_r_* = 905.96
Hexagonal, 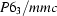

*a* = 6.0880 (2) Å
*c* = 14.1049 (6) Å
*V* = 452.74 (3) Å^3^
*Z* = 2
Mo *K*α radiation
μ = 18.76 mm^−1^
*T* = 293 (2) K
0.06 × 0.04 × 0.03 mm

#### Data collection

Rigaku R-AXIS RAPID diffractometer
Absorption correction: numerical (*NUMABS*; Higashi, 1999 ▶) *T*_min_ = 0.524, *T*_max_ = 0.801
3945 measured reflections
230 independent reflections
215 reflections with *I* > 2σ(*I*)
*R*_int_ = 0.072

#### Refinement

*R*[*F* ^2^ > 2σ(*F* ^2^)] = 0.018
*wR*(*F* ^2^) = 0.041
*S* = 1.13
230 reflections
28 parameters
1 restraint
Δρ_max_ = 0.73 e Å^−3^
Δρ_min_ = −1.81 e Å^−3^

### 

Data collection: *PROCESS-AUTO* (Rigaku/MSC, 2005 ▶); cell refinement: *PROCESS-AUTO*; data reduction: *CrystalStructure* (Rigaku/MSC, 2005 ▶); program(s) used to solve structure: *SHELXS97* (Sheldrick, 2008 ▶); program(s) used to refine structure: *SHELXL97* (Sheldrick, 2008 ▶); molecular graphics: *VESTA* (Momma & Izumi, 2008 ▶); software used to prepare material for publication: *SHELXL97*.

## Supplementary Material

Crystal structure: contains datablocks global, I. DOI: 10.1107/S1600536808030572/mg2057sup1.cif

Structure factors: contains datablocks I. DOI: 10.1107/S1600536808030572/mg2057Isup2.hkl

Additional supplementary materials:  crystallographic information; 3D view; checkCIF report

## Figures and Tables

**Table:** 

Ba1—O1	2.918 (3)
Ba1—O2	3.051 (6)
Sn1—O3	2.068 (3)
Sn2—O1	2.040 (3)
Sn2—O2	2.127 (3)
Co2—O2	1.969 (4)
Co2—O3	2.437 (6)

**Table:** 

O3—Sn1—O3^iv^	180
O3—Sn1—O1^v^	94.67 (11)
O3^iv^—Sn1—O1^v^	85.33 (11)
O1^ii^—Sn2—O1	101.38 (11)
O1^ii^—Sn2—O2	163.32 (12)
O1—Sn2—O2	89.07 (8)
O2^v^—Co2—O2^iii^	120
O2^v^—Co2—O3	90
O3—Co2—O3^i^	180

Symmetry codes: (i) 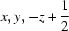; (ii) 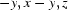; (iii) 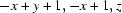; (iv) 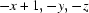; (v) 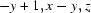.

**Fig. 1.**
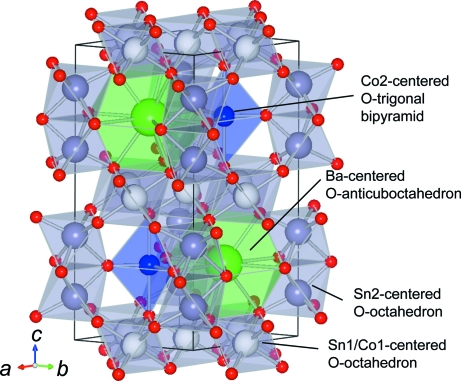
Structure of BaCo2Sn4O11 in terms of cation-centered oxygen polyhedra.

**Fig. 2.**
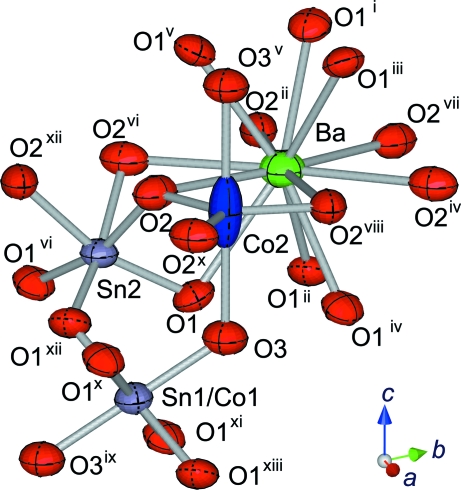
The O-atom coordination around the cation sites of BaCo2Sn4O11 (symmetry codes as in Table 1). Displacement ellipsoids are drawn at the 99% probability level.

## supplementary crystallographic information

### Comment

BaTi_2_Fe_4_O_11_ crystallizes in a six-layer hexagonal structure (*R*-type, space group *P*6_3_/*mmc*) (Haberey & Velicescu, 1974; Obradors *et al.*, 1983; Cadée & Ijdo, 1984; Sosnowska *et al.*, 1996) that is adopted by many multinary iron oxides and transition metal oxides exhibiting complex magnetic behavior (Cadée & Ijdo, 1984; Kanke *et al.*, 1992; Martínez *et al.*, 1993; Foo *et al.*, 2006). BaCo_2_Sn_4_O_11_ has been indicated to be the end member in a solid solution series BaFe_4-2x_Sn_2+_*_x_*Co*_x_*O_11_, where the distribution of Fe, Co, and Sn cations was determined by combined powder X-ray and neutron diffraction (Martínez *et al.*, 1993). However, no crystallographic information (cell parameters and atomic positions) was reported except for the Co and Sn site occupancies in BaCo_2_Sn_4_O_11_. The present paper reports the detailed structure of BaCo_2_Sn_4_O_11_ determined by single-crystal X-ray diffraction.

The structure of BaCo_2_Sn_4_O_11_ can be described in terms of cation-centered oxygen polyhedra (Fig. 1). The disorder within the octahedral 6*g* site (occupancies of 0.664 (7) Sn1 and 0.336 (7) Co1) agrees with results reported by Martínez *et al.* (1993) (0.7 Sn1 and 0.3 Co1). These Sn1/Co1-centered octahedra share edges to form layers perpendicular to the *c* axis. Located between these layers are pillars of two face-sharing Sn2-centered octahedra stacked along the *c* axis. The trigonal bipyramidal 2*d* site is occupied exclusively by Co2 atoms and exhibits a displacement ellipsoid elongated along the *c* direction (Fig. 2). Ba atoms are situated in a 12-fold coordination site (2*c*).

### Experimental

A mixture of BaCO_3_ (99.99%, Wako Pure Chemical Ind.), Co_3_O_4_ (99.95%, Kanto Chemical Co. Inc.), and SnO_2_ (99.9%, Rare Metallic Co. Ltd.) powders in a molar ratio of Ba:Co:Sn = 1:2:4 was ground together and pressed into a pellet. The pellet was placed on a platinum plate and heated in air for 1 h at 1473 K or 1823 K in an electric furnace. The pellet was melted at 1823 K and green transparent single crystals of BaCo_2_Sn_4_O_11_ were obtained. The chemical analysis of the polycrystalline single phase BaCo_2_Sn_4_O_11_ prepared at 1473 K was carried out by inductively coupled plasma (ICP) emission spectrometry for Ba, Co and Sn, and by the He carrier melting-infrared absorption method (TC-436, LECO) for O. The results of the chemical analysis (Ba 14.9 (5), Co 13.8 (5), Sn 52.9 (8), and O 19.1 (8) wt%) agreed with the ideal contents (Ba 15.2, Co 13.4, Sn 51.9, O 19.5 wt%).

The magnetic susceptibility of BaCo_2_Sn_4_O_11_ was measured with a superconducting quantum interference device (SQUID) magnetometer (Quantum Design, MPMS XL) from 5 to 400 K under a magnetic field of 5 kOe. The polycrystalline sample followed the Curie–Weiss law above the Neel temperature (*T*_N_). The *T*_N_ of 7 K, the Weiss temperature (Θ) of -42 K, and the magnetic moment (µ_eff_) of 4.7 µ_B_ per Co measured for the sample were consistent with the values reported by Martínez *et al.* (1993) (*T*_N_ = 9 K, Θ = -44 K, µ_eff_ = 4.4 µ_B_).

### Refinement

In the structure analysis using powder X-ray and neutron diffraction data for BaTi_2_Fe_4_O_11_ and BaSn_2_Fe_4_O_11_ (Obradors *et al.*, 1983; Cadée & Ijdo,1984; Martínez *et al.*, 1993), the 2*d* site of Fe atoms was statistically split into two site (4*f*). We applied this split site model and refined the positional parameter of *z* for the Co2 site, but it converged into 0.250 within an estimated deviation. Thus, we fixed the position at 2*d* site for the final refinement.

### Crystal data

**Table:**

BaCo_2_Sn_4_O_11_	*D*_x_ = 6.646 Mg m^−^^3^
*M**_r_* = 905.96	Mo *K*α radiation, λ = 0.71075 Å
Hexagonal, *P*6_3_/*m**m**c*	Cell parameters from 3536 reflections
Hall symbol: -P 6c 2c	θ = 7.7–54.7°
*a* = 6.0880 (2) Å	µ = 18.76 mm^−^^1^
*c* = 14.1049 (6) Å	*T* = 293 K
*V* = 452.74 (3) Å^3^	Block, green
*Z* = 2	0.06 × 0.04 × 0.03 mm
*F*(000) = 796	

### Data collection

**Table:**

Rigaku R-AXIS RAPID diffractometer	230 independent reflections
Radiation source: fine-focus sealed tube	215 reflections with *I* > 2σ(*I*)
graphite	*R*_int_ = 0.072
Detector resolution: 10.00 pixels mm^-1^	θ_max_ = 27.4°, θ_min_ = 3.9°
ω scans	*h* = −7→7
Absorption correction: numerical (NUMABS; Higashi, 1999)	*k* = −7→7
*T*_min_ = 0.524, *T*_max_ = 0.801	*l* = −18→18
3945 measured reflections	

### Refinement

**Table:**

Refinement on *F*^2^	Primary atom site location: structure-invariant direct methods
Least-squares matrix: full	Secondary atom site location: difference Fourier map
*R*[*F*^2^ > 2σ(*F*^2^)] = 0.018	*w* = 1/[σ^2^(*F*_o_^2^) + 0.8941*P*] where *P* = (*F*_o_^2^ + 2*F*_c_^2^)/3
*wR*(*F*^2^) = 0.041	(Δ/σ)_max_ < 0.001
*S* = 1.13	Δρ_max_ = 0.73 e Å^−^^3^
230 reflections	Δρ_min_ = −1.81 e Å^−^^3^
28 parameters	Extinction correction: SHELXL97 (Sheldrick, 2008), Fc^*^=kFc[1+0.001xFc^2^λ^3^/sin(2θ)]^-1/4^
1 restraint	Extinction coefficient: 0.0041 (4)

### Special details

**Table:**

Refinement. Refinement of *F*^2^ against ALL reflections. The weighted *R*-factor *wR* and goodness of fit *S* are based on *F*^2^, conventional *R*-factors *R* are based on *F*, with *F* set to zero for negative *F*^2^. The threshold expression of *F*^2^ > σ(*F*^2^) is used only for calculating *R*-factors(gt) *etc*. and is not relevant to the choice of reflections for refinement. *R*-factors based on *F*^2^ are statistically about twice as large as those based on *F*, and *R*- factors based on ALL data will be even larger.

### Fractional atomic coordinates and isotropic or equivalent isotropic displacement parameters (Å^2^)

**Table:**

	*x*	*y*	*z*	*U*_iso_*/*U*_eq_	Occ. (<1)
Ba1	0.3333	0.6667	0.2500	0.0147 (2)	
Sn1	0.5000	0.0000	0.0000	0.0112 (3)	0.664 (7)
Co1	0.5000	0.0000	0.0000	0.0112 (3)	0.336 (7)
Sn2	0.0000	0.0000	0.14643 (4)	0.0096 (2)	
Co2	0.6667	0.3333	0.2500	0.0203 (4)	
O1	0.1728 (3)	0.3456 (5)	0.0815 (2)	0.0118 (7)	
O2	0.2933 (8)	0.1466 (4)	0.2500	0.0129 (10)	
O3	0.6667	0.3333	0.0772 (4)	0.0136 (12)	

### Atomic displacement parameters (Å^2^)

**Table:**

	*U*^11^	*U*^22^	*U*^33^	*U*^12^	*U*^13^	*U*^23^
Ba1	0.0169 (3)	0.0169 (3)	0.0103 (4)	0.00844 (15)	0.000	0.000
Sn1	0.0139 (4)	0.0110 (4)	0.0076 (4)	0.0055 (2)	0.00013 (10)	0.0003 (2)
Co1	0.0139 (4)	0.0110 (4)	0.0076 (4)	0.0055 (2)	0.00013 (10)	0.0003 (2)
Sn2	0.0113 (3)	0.0113 (3)	0.0062 (3)	0.00563 (14)	0.000	0.000
Co2	0.0086 (5)	0.0086 (5)	0.0436 (13)	0.0043 (3)	0.000	0.000
O1	0.0139 (12)	0.0132 (17)	0.0080 (17)	0.0066 (8)	0.0015 (6)	0.0030 (13)
O2	0.010 (2)	0.0174 (18)	0.009 (2)	0.0051 (11)	0.000	0.000
O3	0.0159 (18)	0.0159 (18)	0.009 (3)	0.0080 (9)	0.000	0.000

### Geometric parameters (Å, °)

**Table:**

Ba1—O1^i^	2.918 (3)	Sn2—O2^xii^	2.127 (3)
Ba1—O1^ii^	2.918 (3)	Sn2—Sn2^v^	2.9217 (11)
Ba1—O1^iii^	2.918 (3)	Sn2—O3^xvi^	3.6479 (15)
Ba1—O1	2.918 (3)	Sn2—O3^xvii^	3.6479 (15)
Ba1—O1^iv^	2.918 (3)	Sn2—O3	3.6479 (15)
Ba1—O1^v^	2.918 (3)	Sn2—Ba1^xviii^	3.8064 (2)
Ba1—O2^ii^	3.051 (6)	Sn2—Co2^xvi^	3.8064 (2)
Ba1—O2^vi^	3.051 (6)	Co2—O2^x^	1.969 (4)
Ba1—O2^vii^	3.051 (6)	Co2—O2^viii^	1.969 (4)
Ba1—O2^iv^	3.051 (6)	Co2—O2	1.969 (4)
Ba1—O2	3.051 (6)	Co2—O3	2.437 (6)
Ba1—O2^viii^	3.051 (6)	Co2—O3^v^	2.437 (6)
Sn1—O3	2.068 (3)	Co2—Ba1^xix^	3.51492 (11)
Sn1—O3^ix^	2.068 (3)	O1—Co1^viii^	2.074 (2)
Sn1—O1^x^	2.074 (2)	O1—Sn1^viii^	2.074 (2)
Sn1—O1^xi^	2.074 (2)	O1—Co1^vi^	2.074 (2)
Sn1—O1^xii^	2.074 (2)	O1—Sn1^vi^	2.074 (2)
Sn1—O1^xiii^	2.074 (2)	O1—O3^xx^	2.806 (5)
Sn1—Sn1^x^	3.04401 (10)	O1—O3	3.045 (2)
Sn1—Sn1^xiv^	3.04401 (10)	O1—O3^xvi^	3.045 (2)
Sn1—Co1^x^	3.04401 (10)	O1—Co2^xvi^	3.8627 (19)
Sn1—Co1^xiv^	3.04401 (10)	O2—Sn2^v^	2.127 (3)
Sn1—Sn1^xv^	3.04401 (10)	O2—Ba1^xviii^	3.052 (3)
Sn1—Co1^xv^	3.04401 (10)	O2—O3^v^	3.133 (5)
Sn2—O1^vi^	2.040 (3)	O2—O3	3.133 (5)
Sn2—O1	2.040 (3)	O3—Co1^x^	2.068 (3)
Sn2—O1^xii^	2.040 (3)	O3—Sn1^x^	2.068 (3)
Sn2—O2	2.127 (3)	O3—Co1^viii^	2.068 (3)
Sn2—O2^vi^	2.127 (3)	O3—Sn1^viii^	2.068 (3)
			
O1^i^—Ba1—O1^ii^	109.09 (11)	O2—Sn2—Sn2^v^	46.63 (8)
O1^i^—Ba1—O1^iii^	60.31 (9)	O2^vi^—Sn2—Sn2^v^	46.63 (8)
O1^ii^—Ba1—O1^iii^	146.28 (5)	O2^xii^—Sn2—Sn2^v^	46.63 (8)
O1^i^—Ba1—O1	146.28 (5)	O1^vi^—Sn2—O3^xvi^	56.59 (3)
O1^ii^—Ba1—O1	60.31 (9)	O1—Sn2—O3^xvi^	56.59 (3)
O1^iii^—Ba1—O1	146.28 (5)	O1^xii^—Sn2—O3^xvi^	137.79 (12)
O1^i^—Ba1—O1^iv^	146.28 (5)	O2—Sn2—O3^xvi^	122.27 (6)
O1^ii^—Ba1—O1^iv^	60.31 (9)	O2^vi^—Sn2—O3^xvi^	58.89 (11)
O1^iii^—Ba1—O1^iv^	109.09 (12)	O2^xii^—Sn2—O3^xvi^	122.27 (6)
O1—Ba1—O1^iv^	60.31 (9)	Sn2^v^—Sn2—O3^xvi^	105.52 (8)
O1^i^—Ba1—O1^v^	60.31 (9)	O1^vi^—Sn2—O3^xvii^	56.59 (3)
O1^ii^—Ba1—O1^v^	146.28 (5)	O1—Sn2—O3^xvii^	137.79 (12)
O1^iii^—Ba1—O1^v^	60.31 (9)	O1^xii^—Sn2—O3^xvii^	56.59 (3)
O1—Ba1—O1^v^	109.09 (11)	O2—Sn2—O3^xvii^	122.27 (6)
O1^iv^—Ba1—O1^v^	146.28 (5)	O2^vi^—Sn2—O3^xvii^	122.27 (6)
O1^i^—Ba1—O2^ii^	58.59 (5)	O2^xii^—Sn2—O3^xvii^	58.89 (11)
O1^ii^—Ba1—O2^ii^	58.59 (5)	Sn2^v^—Sn2—O3^xvii^	105.52 (8)
O1^iii^—Ba1—O2^ii^	118.76 (5)	O3^xvi^—Sn2—O3^xvii^	113.12 (7)
O1—Ba1—O2^ii^	92.30 (4)	O1^vi^—Sn2—O3	137.79 (12)
O1^iv^—Ba1—O2^ii^	118.76 (5)	O1—Sn2—O3	56.59 (3)
O1^v^—Ba1—O2^ii^	92.30 (5)	O1^xii^—Sn2—O3	56.59 (3)
O1^i^—Ba1—O2^vi^	92.30 (4)	O2—Sn2—O3	58.89 (11)
O1^ii^—Ba1—O2^vi^	92.30 (4)	O2^vi^—Sn2—O3	122.27 (6)
O1^iii^—Ba1—O2^vi^	118.76 (5)	O2^xii^—Sn2—O3	122.27 (6)
O1—Ba1—O2^vi^	58.59 (5)	Sn2^v^—Sn2—O3	105.52 (8)
O1^iv^—Ba1—O2^vi^	118.76 (5)	O3^xvi^—Sn2—O3	113.12 (7)
O1^v^—Ba1—O2^vi^	58.59 (5)	O3^xvii^—Sn2—O3	113.12 (7)
O2^ii^—Ba1—O2^vi^	67.94 (15)	O1^vi^—Sn2—Ba1^xviii^	125.796 (15)
O1^i^—Ba1—O2^vii^	58.59 (5)	O1—Sn2—Ba1^xviii^	125.796 (15)
O1^ii^—Ba1—O2^vii^	58.59 (5)	O1^xii^—Sn2—Ba1^xviii^	49.26 (9)
O1^iii^—Ba1—O2^vii^	92.30 (4)	O2—Sn2—Ba1^xviii^	53.189 (6)
O1—Ba1—O2^vii^	118.76 (5)	O2^vi^—Sn2—Ba1^xviii^	114.06 (8)
O1^iv^—Ba1—O2^vii^	92.30 (4)	O2^xii^—Sn2—Ba1^xviii^	53.189 (6)
O1^v^—Ba1—O2^vii^	118.76 (5)	Sn2^v^—Sn2—Ba1^xviii^	67.432 (8)
O2^ii^—Ba1—O2^vii^	52.06 (15)	O3^xvi^—Sn2—Ba1^xviii^	172.95 (8)
O2^vi^—Ba1—O2^vii^	120.000 (1)	O3^xvii^—Sn2—Ba1^xviii^	69.99 (4)
O1^i^—Ba1—O2^iv^	92.30 (4)	O3—Sn2—Ba1^xviii^	69.99 (4)
O1^ii^—Ba1—O2^iv^	92.30 (4)	O1^vi^—Sn2—Co2^xvi^	76.11 (5)
O1^iii^—Ba1—O2^iv^	58.59 (5)	O1—Sn2—Co2^xvi^	76.11 (5)
O1—Ba1—O2^iv^	118.76 (5)	O1^xii^—Sn2—Co2^xvi^	175.88 (9)
O1^iv^—Ba1—O2^iv^	58.59 (5)	O2—Sn2—Co2^xvi^	94.13 (5)
O1^v^—Ba1—O2^iv^	118.76 (5)	O2^vi^—Sn2—Co2^xvi^	20.80 (8)
O2^ii^—Ba1—O2^iv^	120.0	O2^xii^—Sn2—Co2^xvi^	94.13 (5)
O2^vi^—Ba1—O2^iv^	172.06 (15)	Sn2^v^—Sn2—Co2^xvi^	67.432 (8)
O2^vii^—Ba1—O2^iv^	67.94 (15)	O3^xvi^—Sn2—Co2^xvi^	38.09 (8)
O1^i^—Ba1—O2	118.76 (5)	O3^xvii^—Sn2—Co2^xvi^	123.20 (2)
O1^ii^—Ba1—O2	118.76 (5)	O3—Sn2—Co2^xvi^	123.20 (2)
O1^iii^—Ba1—O2	92.30 (4)	Ba1^xviii^—Sn2—Co2^xvi^	134.863 (15)
O1—Ba1—O2	58.59 (5)	O2^x^—Co2—O2^viii^	120.000 (1)
O1^iv^—Ba1—O2	92.30 (4)	O2^x^—Co2—O2	120.0
O1^v^—Ba1—O2	58.59 (5)	O2^viii^—Co2—O2	120.0
O2^ii^—Ba1—O2	120.0	O2^x^—Co2—O3	90.0
O2^vi^—Ba1—O2	52.06 (15)	O2^viii^—Co2—O3	90.000 (1)
O2^vii^—Ba1—O2	172.06 (15)	O2—Co2—O3	90.0
O2^iv^—Ba1—O2	120.0	O2^x^—Co2—O3^v^	90.0
O1^i^—Ba1—O2^viii^	118.76 (5)	O2^viii^—Co2—O3^v^	90.0
O1^ii^—Ba1—O2^viii^	118.76 (5)	O2—Co2—O3^v^	90.0
O1^iii^—Ba1—O2^viii^	58.59 (5)	O3—Co2—O3^v^	180.0
O1—Ba1—O2^viii^	92.30 (4)	O2^x^—Co2—Ba1^xix^	60.0
O1^iv^—Ba1—O2^viii^	58.59 (5)	O2^viii^—Co2—Ba1^xix^	60.0
O1^v^—Ba1—O2^viii^	92.30 (4)	O2—Co2—Ba1^xix^	180.0
O2^ii^—Ba1—O2^viii^	172.06 (15)	O3—Co2—Ba1^xix^	90.0
O2^vi^—Ba1—O2^viii^	120.0	O3^v^—Co2—Ba1^xix^	90.0
O2^vii^—Ba1—O2^viii^	120.0	Sn2—O1—Co1^viii^	126.84 (8)
O2^iv^—Ba1—O2^viii^	52.06 (15)	Sn2—O1—Sn1^viii^	126.84 (8)
O2—Ba1—O2^viii^	67.94 (15)	Co1^viii^—O1—Sn1^viii^	0.0
O3—Sn1—O3^ix^	180.0 (3)	Sn2—O1—Co1^vi^	126.84 (8)
O3—Sn1—O1^x^	94.67 (11)	Co1^viii^—O1—Co1^vi^	94.45 (12)
O3^ix^—Sn1—O1^x^	85.33 (11)	Sn1^viii^—O1—Co1^vi^	94.45 (12)
O3—Sn1—O1^xi^	85.33 (11)	Sn2—O1—Sn1^vi^	126.84 (8)
O3^ix^—Sn1—O1^xi^	94.67 (11)	Co1^viii^—O1—Sn1^vi^	94.45 (12)
O1^x^—Sn1—O1^xi^	180.00 (14)	Sn1^viii^—O1—Sn1^vi^	94.45 (12)
O3—Sn1—O1^xii^	94.67 (11)	Co1^vi^—O1—Sn1^vi^	0.0
O3^ix^—Sn1—O1^xii^	85.33 (11)	Sn2—O1—O3^xx^	153.78 (16)
O1^x^—Sn1—O1^xii^	89.97 (16)	Co1^viii^—O1—O3^xx^	47.25 (6)
O1^xi^—Sn1—O1^xii^	90.03 (16)	Sn1^viii^—O1—O3^xx^	47.25 (6)
O3—Sn1—O1^xiii^	85.33 (11)	Co1^vi^—O1—O3^xx^	47.25 (6)
O3^ix^—Sn1—O1^xiii^	94.67 (11)	Sn1^vi^—O1—O3^xx^	47.25 (6)
O1^x^—Sn1—O1^xiii^	90.03 (16)	Sn2—O1—Ba1	98.77 (12)
O1^xi^—Sn1—O1^xiii^	89.97 (16)	Co1^viii^—O1—Ba1	102.93 (9)
O1^xii^—Sn1—O1^xiii^	180.00 (14)	Sn1^viii^—O1—Ba1	102.93 (9)
O3—Sn1—Sn1^x^	42.60 (9)	Co1^vi^—O1—Ba1	102.93 (9)
O3^ix^—Sn1—Sn1^x^	137.40 (9)	Sn1^vi^—O1—Ba1	102.93 (9)
O1^x^—Sn1—Sn1^x^	91.55 (7)	O3^xx^—O1—Ba1	107.45 (11)
O1^xi^—Sn1—Sn1^x^	88.45 (7)	Sn2—O1—O3	89.42 (7)
O1^xii^—Sn1—Sn1^x^	137.22 (6)	Co1^viii^—O1—O3	42.59 (8)
O1^xiii^—Sn1—Sn1^x^	42.78 (6)	Sn1^viii^—O1—O3	42.59 (8)
O3—Sn1—Sn1^xiv^	137.40 (9)	Co1^vi^—O1—O3	136.98 (15)
O3^ix^—Sn1—Sn1^xiv^	42.60 (9)	Sn1^vi^—O1—O3	136.98 (15)
O1^x^—Sn1—Sn1^xiv^	88.45 (7)	O3^xx^—O1—O3	89.84 (10)
O1^xi^—Sn1—Sn1^xiv^	91.55 (7)	Ba1—O1—O3	91.63 (10)
O1^xii^—Sn1—Sn1^xiv^	42.78 (6)	Sn2—O1—O3^xvi^	89.42 (7)
O1^xiii^—Sn1—Sn1^xiv^	137.22 (6)	Co1^viii^—O1—O3^xvi^	136.98 (15)
Sn1^x^—Sn1—Sn1^xiv^	180.0	Sn1^viii^—O1—O3^xvi^	136.98 (15)
O3—Sn1—Co1^x^	42.60 (9)	Co1^vi^—O1—O3^xvi^	42.59 (8)
O3^ix^—Sn1—Co1^x^	137.40 (9)	Sn1^vi^—O1—O3^xvi^	42.59 (8)
O1^x^—Sn1—Co1^x^	91.55 (7)	O3^xx^—O1—O3^xvi^	89.84 (10)
O1^xi^—Sn1—Co1^x^	88.45 (7)	Ba1—O1—O3^xvi^	91.63 (10)
O1^xii^—Sn1—Co1^x^	137.22 (6)	O3—O1—O3^xvi^	176.68 (18)
O1^xiii^—Sn1—Co1^x^	42.78 (6)	Sn2—O1—Co2	73.06 (6)
Sn1^x^—Sn1—Co1^x^	0.0	Co1^viii^—O1—Co2	76.66 (3)
Sn1^xiv^—Sn1—Co1^x^	180.0	Sn1^viii^—O1—Co2	76.66 (3)
O3—Sn1—Co1^xiv^	137.40 (9)	Co1^vi^—O1—Co2	157.83 (12)
O3^ix^—Sn1—Co1^xiv^	42.60 (9)	Sn1^vi^—O1—Co2	157.83 (12)
O1^x^—Sn1—Co1^xiv^	88.45 (7)	O3^xx^—O1—Co2	120.06 (6)
O1^xi^—Sn1—Co1^xiv^	91.55 (7)	Ba1—O1—Co2	60.56 (4)
O1^xii^—Sn1—Co1^xiv^	42.78 (6)	O3—O1—Co2	39.11 (11)
O1^xiii^—Sn1—Co1^xiv^	137.22 (6)	O3^xvi^—O1—Co2	143.08 (14)
Sn1^x^—Sn1—Co1^xiv^	180.0	Sn2—O1—Co2^xvi^	73.06 (6)
Sn1^xiv^—Sn1—Co1^xiv^	0.0	Co1^viii^—O1—Co2^xvi^	157.83 (12)
Co1^x^—Sn1—Co1^xiv^	180.0	Sn1^viii^—O1—Co2^xvi^	157.83 (12)
O3—Sn1—Sn1^xv^	137.40 (9)	Co1^vi^—O1—Co2^xvi^	76.66 (3)
O3^ix^—Sn1—Sn1^xv^	42.60 (9)	Sn1^vi^—O1—Co2^xvi^	76.66 (3)
O1^x^—Sn1—Sn1^xv^	42.78 (6)	O3^xx^—O1—Co2^xvi^	120.06 (6)
O1^xi^—Sn1—Sn1^xv^	137.22 (6)	Ba1—O1—Co2^xvi^	60.56 (4)
O1^xii^—Sn1—Sn1^xv^	88.45 (7)	O3—O1—Co2^xvi^	143.08 (14)
O1^xiii^—Sn1—Sn1^xv^	91.55 (7)	O3^xvi^—O1—Co2^xvi^	39.11 (11)
Sn1^x^—Sn1—Sn1^xv^	120.0	Co2—O1—Co2^xvi^	104.01 (7)
Sn1^xiv^—Sn1—Sn1^xv^	60.0	Co2—O2—Sn2	136.63 (8)
Co1^x^—Sn1—Sn1^xv^	120.0	Co2—O2—Sn2^v^	136.63 (8)
Co1^xiv^—Sn1—Sn1^xv^	60.0	Sn2—O2—Sn2^v^	86.75 (15)
O3—Sn1—Co1^xv^	137.40 (9)	Co2—O2—Ba1^xviii^	86.03 (8)
O3^ix^—Sn1—Co1^xv^	42.60 (9)	Sn2—O2—Ba1^xviii^	92.88 (5)
O1^x^—Sn1—Co1^xv^	42.78 (6)	Sn2^v^—O2—Ba1^xviii^	92.88 (5)
O1^xi^—Sn1—Co1^xv^	137.22 (6)	Co2—O2—Ba1	86.03 (8)
O1^xii^—Sn1—Co1^xv^	88.45 (7)	Sn2—O2—Ba1	92.88 (5)
O1^xiii^—Sn1—Co1^xv^	91.55 (7)	Sn2^v^—O2—Ba1	92.88 (5)
Sn1^x^—Sn1—Co1^xv^	120.0	Ba1^xviii^—O2—Ba1	172.06 (15)
Sn1^xiv^—Sn1—Co1^xv^	60.0	Co2—O2—O3^v^	51.07 (9)
Co1^x^—Sn1—Co1^xv^	120.0	Sn2—O2—O3^v^	172.31 (15)
Co1^xiv^—Sn1—Co1^xv^	60.0	Sn2^v^—O2—O3^v^	85.56 (7)
Sn1^xv^—Sn1—Co1^xv^	0.0	Ba1^xviii^—O2—O3^v^	87.51 (5)
O1^vi^—Sn2—O1	101.38 (11)	Ba1—O2—O3^v^	87.51 (5)
O1^vi^—Sn2—O1^xii^	101.38 (11)	Co2—O2—O3	51.07 (9)
O1—Sn2—O1^xii^	101.38 (11)	Sn2—O2—O3	85.56 (7)
O1^vi^—Sn2—O2	163.32 (12)	Sn2^v^—O2—O3	172.31 (15)
O1—Sn2—O2	89.07 (8)	Ba1^xviii^—O2—O3	87.51 (5)
O1^xii^—Sn2—O2	89.07 (8)	Ba1—O2—O3	87.51 (5)
O1^vi^—Sn2—O2^vi^	89.07 (8)	O3^v^—O2—O3	102.13 (17)
O1—Sn2—O2^vi^	89.07 (8)	Sn1—O3—Co1^x^	94.80 (17)
O1^xii^—Sn2—O2^vi^	163.32 (12)	Sn1—O3—Sn1^x^	94.80 (17)
O2—Sn2—O2^vi^	78.03 (12)	Co1^x^—O3—Sn1^x^	0.0
O1^vi^—Sn2—O2^xii^	89.07 (8)	Sn1—O3—Co1^viii^	94.80 (17)
O1—Sn2—O2^xii^	163.32 (12)	Co1^x^—O3—Co1^viii^	94.80 (17)
O1^xii^—Sn2—O2^xii^	89.07 (8)	Sn1^x^—O3—Co1^viii^	94.80 (17)
O2—Sn2—O2^xii^	78.03 (12)	Sn1—O3—Sn1^viii^	94.80 (17)
O2^vi^—Sn2—O2^xii^	78.03 (12)	Co1^x^—O3—Sn1^viii^	94.80 (17)
O1^vi^—Sn2—Sn2^v^	116.69 (9)	Sn1^x^—O3—Sn1^viii^	94.80 (17)
O1—Sn2—Sn2^v^	116.69 (9)	Co1^viii^—O3—Sn1^viii^	0.0
O1^xii^—Sn2—Sn2^v^	116.69 (9)		

Symmetry codes: (i) −*x*+*y*, −*x*+1, −*z*+1/2; (ii) −*x*+*y*, −*x*+1, *z*; (iii) −*y*+1, *x*−*y*+1, −*z*+1/2; (iv) −*y*+1, *x*−*y*+1, *z*; (v) *x*, *y*, −*z*+1/2; (vi) −*y*, *x*−*y*, *z*; (vii) *x*, *y*+1, *z*; (viii) −*x*+*y*+1, −*x*+1, *z*; (ix) −*x*+1, −*y*, −*z*; (x) −*y*+1, *x*−*y*, *z*; (xi) *y*, −*x*+*y*, −*z*; (xii) −*x*+*y*, −*x*, *z*; (xiii) *x*−*y*+1, *x*, −*z*; (xiv) −*y*, *x*−*y*−1, *z*; (xv) −*x*+*y*+1, −*x*, *z*; (xvi) *x*−1, *y*, *z*; (xvii) *x*−1, *y*−1, *z*; (xviii) *x*, *y*−1, *z*; (xix) *x*+1, *y*, *z*; (xx) −*x*+1, −*y*+1, −*z*.
